# Application of the Single Use Negative Pressure Wound Therapy Device (PICO) on a Heterogeneous Group of Surgical and Traumatic Wounds

**Published:** 2014-04-28

**Authors:** Caroline Payne, Daren Edwards

**Affiliations:** The Royal London Hospital, Barts Health NHS Trust

**Keywords:** plastic surgery, wound management, negative pressure wound dressing, single use device, early patient discharge

## Abstract

**Objectives:** Traumatic wounds and surgery inherently have their complications. Localized infections, wound dehiscence, and excessive wound leakage can be devastating to the patient with a prolonged recovery, but it is also costly to the hospital with an increased length of stay, extra workload, and dressing changes. The single use PICO (Smith and Nephew Healthcare, Hull, United Kingdom) negative pressure wound therapy (NPWT) dressing has revolutionized our management of various acute, chronic, and high output wounds. It requires fewer dressing changes than conventional practice, is used in the outpatient setting, and is a necessary adjuvant therapy to hasten wound healing. **Aims:** To observe the efficacy of the PICO vacuum-assisted healing within a cost improvement programme. **Settings:** Plastic surgery department, Royal London Hospital. **Materials and Methods:** Twenty-one patients with a diversity of postoperative or posttraumatic wounds were considered suitable for PICO application and treated totally on an outpatient basis once the PICO dressing was applied. All wounds were then subjected to continued PICO dressings until healed. **Results:** All patients tolerated the PICO well with no dressing failure or failure to comply. The number of dressings per patient ranged from 1 to 7. The cost per patient of treatment ranged from £120 to £1578. Estimated cost of all PICO dressing for 21 patients including plastic surgery dressing clinic appointments = £13,345. Median length of treatment to healing (days) = 16; standard deviation = 9.5. Eight patients would have had an inpatient bed stay with conventional therapy, total 24 bed days saved at Bartshealth @£325 per day. **Conclusions:** The outpatient application of a disposable NPWT can benefit a wide range of clinical wounds that optimizes patient care, promotes rapid wound healing, and importantly helps manage costs.

The intrinsic nature of surgery in all specialties means that there can be adverse events associated with the operation. These events vary to those associated specifically to the type of surgery performed, to the universal problematic complications of wound dehiscence and localized infection. Surgical site infection (SSI) is costly not only in fiscal terms to the admitting hospital but primarily to the patient in regard to physical and psychological well being, extended length of stay, and repeated, usually painful, dressing changes to the area. Surgical site infections account for 15.7% of hospital-acquired infections in hospital inpatients (Health Protection Agency, SSI prevalence data for 2011),^1^ and at least 5% of patients undergoing a surgical procedure develop a SSI.^2^ In 2008, this alone increased the costs of individual patient care by £814 to £6626 depending on the type of surgery and the severity of the infection (overall cost to the NHS exceeds £90 m), a major part of this cost is the extra inpatient bed stay. This has led to an integrated management approach and specific care pathways, which is now at the forefront of hospital prevention plans and includes assessment of wound dressings.

The application of negative pressure wound therapy (NPWT) to an operative site is now a commonplace. Negative pressure wound therapy was popularized after reports by Morykwas et al^3^ and their research on wounds treated by topical negative pressure in animal and clinical studies.^4^ The standard porous polyurethane foam system was commercialized by KCI (Kinetic Concepts Incorporation, San Antonio, Texas) into the VAC system. Negative pressure wound therapy is a generic technology with wide range of devices available in the commercial market that provide variable topical negative pressure, wound contact layer, and portability. Negative pressure wound therapy has several modes of action on wound beds including angiogenesis, stimulation of growth of granulation tissue,^5^ evacuation of wound exudate, decreased bacterial bioburden,^6^ and wound contraction.^7^ There is a strength of evidence of its clinical efficacy with more than 1000 reviewed publications, so it has become widely used in the management of a variety of surgical beds either to prepare it for further a procedure^8^^,^^9^ or to promote the healing of a definitive procedure.^10^^,^^11^ It is frequently used in circumstances where it is desired to expedite the normal healing process after uncomplicated surgery or after trauma.^12^^-^14

In many circumstances, this group of patients can be managed on the short stay admission wards to then enable most of their postoperative recovery in their own home. Advances in surgical technique have helped achieve these goals in the form of minimally invasive procedures, robotics, and better application of local anesthetics to block the operative site foregoing the need for recovery from a general anesthetic.^15^ The confounding factor is that although the surgery can be done as a day case, the length of stay can be prolonged due to application of a nonportable NPWT device.

To compliment this holistic patient episode in the short stay surgical unit, there is the necessity to develop reliable and advanced wound dressings that promote healing, are comfortable to the patient, require minimal changes, and can be totally performed as an outpatient. The use of NPWT in the community is expanding with the advent of smaller, more portable devices. In Bart's Health NHS Trust, we have found obstacles to the use of community NPWT in the past. There has been a combination of reasons for this, including lack of funding and health care professionals’ unfamiliarity with the usage of NPWT devices. The benefits of portable NPWT devices can be clearly seen. In particular, the PICO system retains the benefits of many ambulatory systems with the addition of its own specific advantages.

We have found that the PICO system achieves all designated treatment goals and have reviewed patients in our department to assess the overall advantages of the PICO system and the fiscal burden.

## MATERIAL AND METHODS

### Study design

A retrospective and ongoing case study evaluation of the pocket-sized ambulatory PICO (Smith and Nephew Healthcare, Hull, United Kingdom) NWPT system was carried out at the Royal London Hospital (RLH) plastic surgery department and in the plastic surgery dressing clinic (PDC). All patients provided written consent to access confidential notes and photographs. The study analyzed patient wounds, number of dressings, estimated cost per patient, and in some cases an estimate of financial gain compared to inpatient hospitalization. Twenty-two patients were identified and 1 refused consent for release of material. There is ongoing patient follow-up, maximum 22 months from time of healed wound.

### NPWT device

The compact and ambulatory PICO NPWT system (Smith and Nephew) has been used on an outpatient basis in the plastic surgery department over the last year. The unit is very compact and ultra lightweight—this makes it very practical for ambulatory patients. Patients found it very straightforward to use—there is no canister (as the dressing can hold up to 300 mL of exudate) or consumable to replace. The device is packaged for single use and therefore does not need returning or sterilization. The battery pack retains its charge for 7 days delivering a continuous negative pressure of −70 mm Hg, which is within the evidence-based therapeutic range. The silicone wound contact layer does not lead to in-growth of granulation tissue into the dressing material. This helps to alleviate patient discomfort on dressing changes and does not disrupt the re-epithelization process.

### Costs

The cost per PICO is £120 unit (1 battery pack with batteries and 2 dressings). Cost of 1 PDC visit @£82 with no compensated travel costs, or with travel cost (up to £76) @£171 per visit. Inpatient bed stay cost at RLH @£325 per day (figure from finance department, RLH). The vacuum-assisted closure system (VAC, KCI) costs RLH £140 for a 3-day hire and sponge. Estimated cost of a 3-day inpatient stay with a VAC post a split-thickness skin graft (STSG) to a wound bed at RLH estimated at £1115.

### Case studies

In the RLH plastic surgery department, 21 patients (12 women and 9 man) were identified, 8 retrospectively and 13 prospectively as having or requiring PICO application (Table 1). Ten applications were for traumatic wounds and 11 for postoperative complications. Their progress was followed throughout their treatment identifying the end point as healed with no further intervention needing PICO therapy. Six patients were considered for case studies due to the significance of their wound and use of the PICO in exceptional circumstances.

### Case study 1

**Figure 1:**
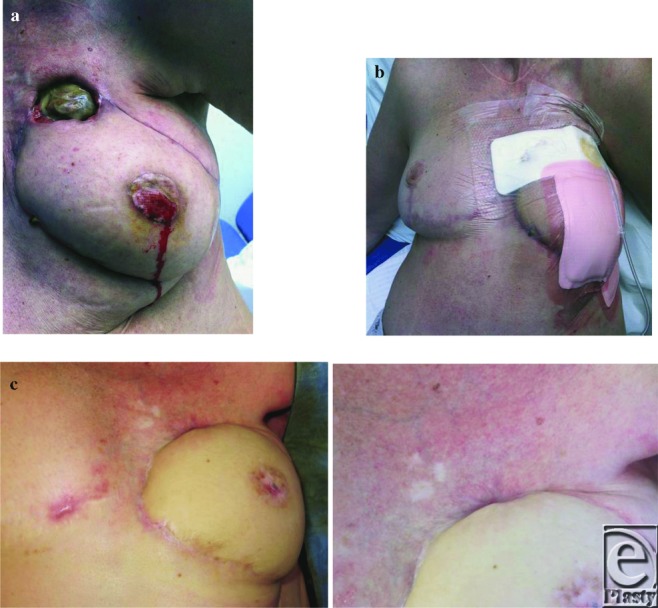
(a) Wound on first presentation in surgical outpatients department. Excessive amount of exudate from radiation damaged skin breakdown and fat necrosis. Bleeding from the nipple area as eschar debrided. (b) First application of the PICO with Allevyn foam to cover the nipple area. Dressing pad shows exudate leaking into the dressing after the first application. (c) Area after 7 PICO applications, June 2012.

A 61-year-old woman with a history of neuroblastoma treated as a child with mantel radiation and to the left chest wall. Consequential left breast hypoplasia was reconstructed with an implant at the age of 22 years requiring several capsulotomies and exchanges. On referral had a grade-4 capsular contracture and breast distortion. Past Medical History (PMH): Thyroidectomy and severe radiation-induced pulmonary fibrosis. Her surgical option for reconstruction was with a pedicle TRAM flap, performed on February 23, 2012. The wound broke down on the superior medial aspect of the breast with fat necrosis due to radiation damage (Fig 1a). A PICO dressing was applied on March 19, 2012, in PDC to manage wound, odor, and exudate (Fig 1b).

Reviewed on April 20, 2012, and wound exudate was dissipated completely and changed to AQUACEL and DuoDERM (ConvaTec, Skillman, New Jersey) until healed (Fig 1c). Seven complete changes of PICO were done as an outpatient in 37 days; 9 PDC visits; grand total = £1578.

### Case study 2

**Figure 2:**
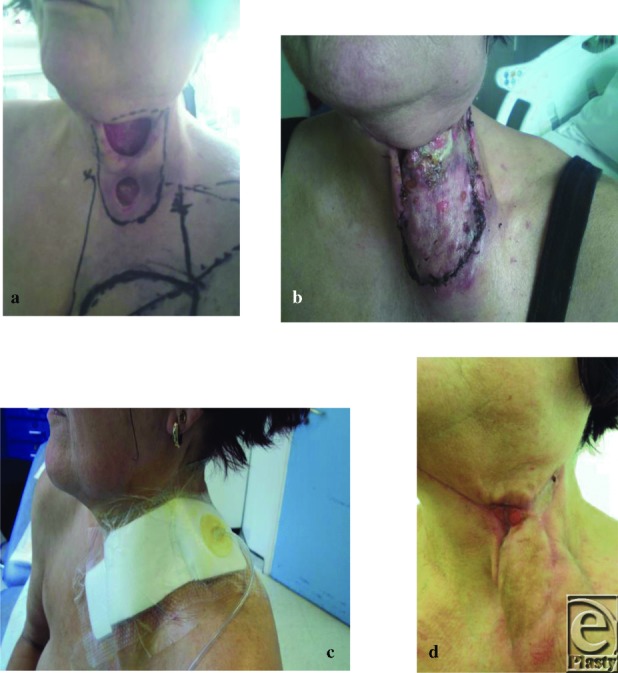
(a) Wound prior to pectoralis major flap cover. (b) Postoperative stage following flap repair and skin graft coverage. Wound shows first stages of breakdown along proximal edges on July 14, 2012. (c) First application of PICO on July 18, 2012. Extra OpSite around the dressing edges to facilitate neck movement. PICO changed twice weekly for 2 weeks to monitor exudate management and observe wound coverage. (d) Wound on August 16, 2012. Decision made to discontinue PICO and manage conservatively and treat areas of over granulation with nitrate.

A 59-year-old woman presented with dehisced wound following breakdown of a left neck dissection (June 2012) after excision of squamous cell carcinoma of the palate (Fig 2a). PMH: Known alcohol-related cirrhosis and ex-smoker. Readmitted for wound care and exudate management. Wound size at this time was 7 × 5 cm^2^. Exudate leakage was causing excoriation of surrounding skin. Wound care plan at the time was packing with AQUACEL (ConvaTec) and Iodoflex (Smith and Nephew) to slough areas and Mepilex border (Mölnlycke Health Care, Gothenburg, Sweden) and the dressing to be changed according to the level of exudate or daily. Wound initially responded well, but further deterioration required the defect to be covered with a pectoralis major flap and skin graft (Fig 2b) in July 2012. While an inpatient, failure of the superior aspect of the graft with further dehiscence of the wound edge occurred. After wound review, a PICO dressing was applied (Fig 2c) and the patient discharged home. Total time with PICO: 29 days; 5 complete changes at the Royal London and 9 visits to PDC; grand total = £1338 (Fig 2d).

### Case study 3

**Figure 3:**
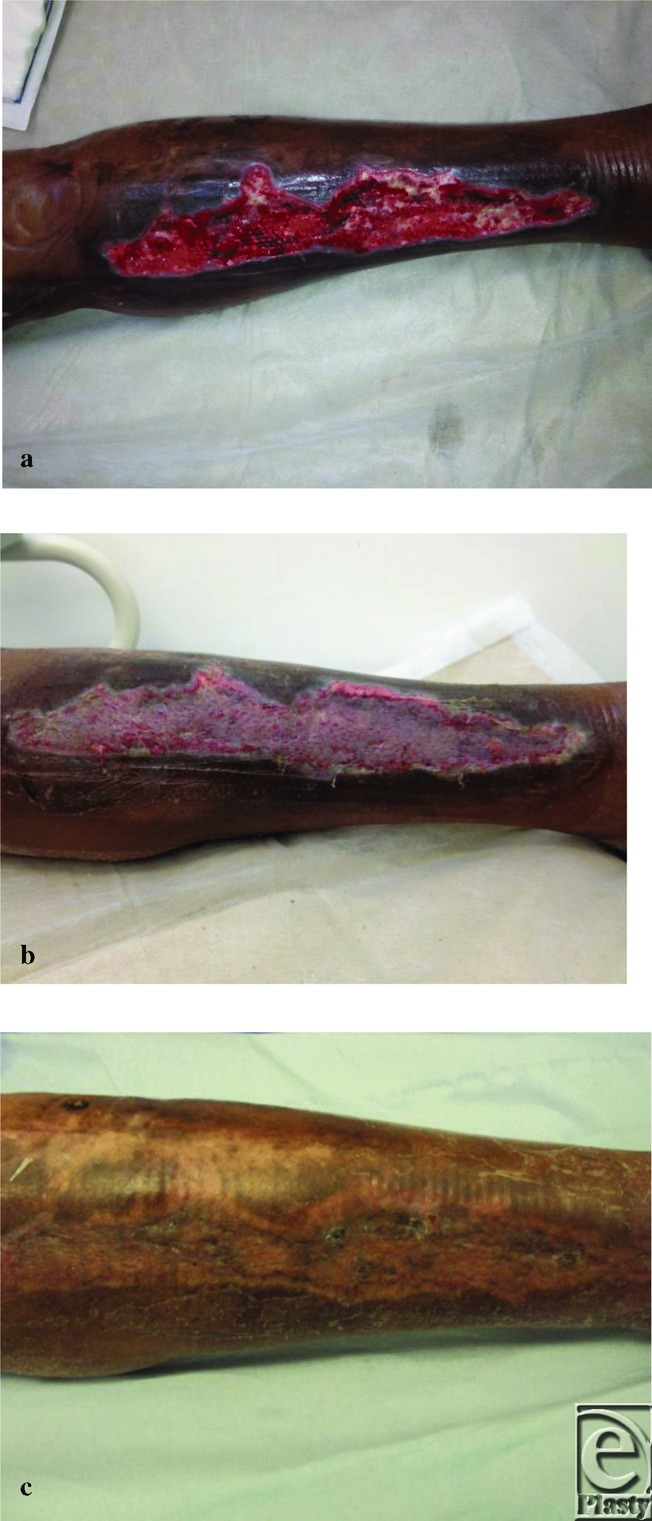
(a) Wound at initial debridement. (b) At the time of first SSG check and reapplication of PICO. (c) Healed fully by October 2012.

A 73-year old man presented. PMH: Ischemic heart disease, type 1 diabetes, peripheral vascular disease, and active smoker. Medication included aspirin 75 mg and clopidogrel 75 mg. He underwent a coronary artery bypass graft in June 2012 but developed a wound breakdown on medial aspect left lower leg from saphenous vein graft site. Discharged home with dressings to left lower leg treated by a district nurse. Leg wound required active infection washout and debridement on July 19 and application of VAC (KCI) as inpatient. Referred to plastic surgery on July 27 for wound management and discharged the same day with no VAC (KCI). and simple dressings. Admitted as a day case, August 2, for wound debridement and application of PICO (Fig 3a) with only the aspirin stopped for 6 days prior to surgery. After 1 change of PICO, admitted for split skin graft and discharged same day. PICO discontinued 2 weeks after split skin graft (SSG) (Fig 3b). Patient continued to smoke in his postoperative phase but was healed by the beginning of October (Fig 3c). Total changes of PICO: £120 × 4 = £480, 4 visits to PDC for PICO change; grand total £808.

### Case study 4

**Figure 4:**
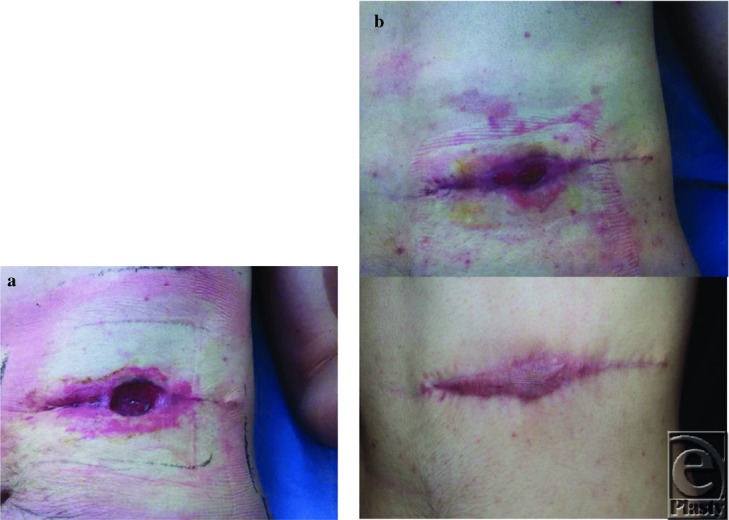
(a) Central wound dehiscence left flank. PICO applied on 13 July. (b) Wound nearly closed by August 7. No further PICO dressing from this time and fully healed by end August.

A 26-year-old man with dermatofibroma protuberans to the lower chest wall 3 × 3 cm^2^ presented. PMH: No significant PMH and a nonsmoker. Operated on May 24, 2012, for a wide local excision and direct closure. Patient was advised that the closure would be tight and would need to lie flexed to prevent dehiscence. The excision margins were clear but he continued coming to plastic dressing clinic twice weekly with central wound dehiscence (Fig 4a). PICO then applied to wound on July 13. Wound healed with no grafting (Fig 4b) with total 10 visits to clinic at the rate of £82 per visit, so £82 × 10 = £820; PICO dressing changes 4 times at the rate of £120, so 4 × £120 = £480; grand total = £1300.

### Case study 5

**Figure 5:**
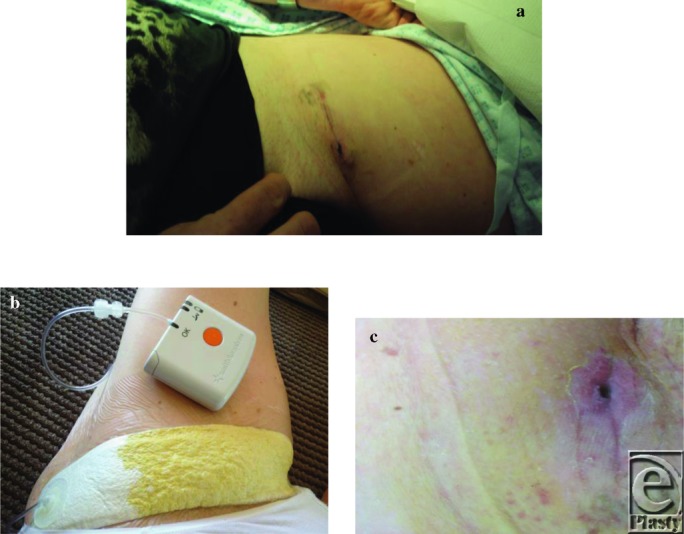
(a) Initial view of the left groin showing the site of SLNB and the difficulties in covering the wound area with adequate dressings. The dehiscence was not a concern but the copious amount of seroma leakage. (b) Dressing 1 week later with exudate manageable. PICO dressing had been changed at 3 days by general practitioner, then returning to PDC at RLH for weekly review. (c) SLNB site 5 weeks later. Seroma fluid now dissipated, small exit wound. Decision made to stop PICO and return to conventional dressings. Wound healed 2 weeks later.

A referral of a 43-year-old woman after a previous excision biopsy of 1.2 mm (pT2A) melanoma left calf in April 2012 presented. She had a day-case wide local excision and a positive sentinel lymph node biopsy (SLNB) requiring a further lymph node dissection in July 2012. At 1 week, she returned to PDC complaining of excessive seroma leakage (up to 1 L a day) from the SLNB site (Fig 5a). She was initially treated with Mepilex Border (Mölnlycke Health Care), which would not absorb the amount of seroma draining. The patient was unable to cope and could not return to work. She wanted to avoid readmission as she travelled from more than 70 miles away. A PICO dressing was applied and secured using barrier skin preparations (Cavilon, 3M Healthcare, Loughborough, UK) (Fig 5b). The initial dressing required change within 24 hours but the next was 2 to 3 days later. After 2 weeks, PICO dressings were performed weekly. Total time with PICO: 31 days; 5 complete changes at the Royal London Surgical Outpatients Department; 2 changes taking place at home; total £846 until considered healed (Fig 5c).

### Case study 6

A 57-year-old lady, diabetic, nonsmoker underwent a left-sided neck dissection for metastatic melanoma on November 17, 2011. On postoperative day 2, she underwent reexploration due to dramatic chyle leak. She continued having high output drainage, average 2550 mL per day, even with very low fat diet and control of hyponatremia. She started total parental nutrition (TPN) on the November 25 but continued with high output up to 5230 mL a day. She had an omohyoid and hemi-SCM muscle flap to cover the leak and sealed with TISSEEL (Baxter Healthcare, Newbury, UK) on December 1. The TPN was stopped and normal diet resumed on December 7, but the fluid output (biochemically serous fluid) continued to a maximum of 300 mL daily. After deciding to remove the drain with an output between 160 and 220 mL per 24 hours and applying PICO over the wound site on December 11, she was discharged the same day after being in hospital for 24 days. PICO dressing needed replacing as soaked every 24 hours until December 16 and then the dressing lasted 4 days after which it was applied weekly for 2 weeks. During the weekly PICO application, the dressing acquired minimal exudate. PICO stopped with no further drainage from the left neck on January 4, 2012. Total 8 visits to clinic, so £82 × 8 = £656; PICO dressing changes 5 times at the rate of £120, so 5 × £120 = £600; grand total = £1256.

## RESULTS

Twenty-one patients were treated with the PICO only on an outpatient basis (Table 1). Once the PICO had been applied on the ward, the patient was discharged home. Ten patients required an STSG to a wound bed within the management plan, with no loss of graft visualized in any patient. All patients tolerated the PICO well with no dressing failure or failure to comply. The number of PICO packs per patient ranged from 1 to 7 (7 PICO packs contain 14 dressings). This means that even though the patient had a visit to the clinic, it did not always require a new PICO to be opened as the 2 AA lithium batteries supplied last up to 7 days. The cost per patient of treatment, with clinic visits ranged from £120 to £1578. The total cost of all PICO treatments, including clinic appointments = £13,345 (1 × PICO + PDC = £202). Median length of treatment to healing = 16.25 days, standard deviation: 9.5, range: 7 to 31. The minimum time for follow-up was 8 weeks and maximum was 22 months. Nine patients were skin grafted and sent home the same day. The departmental practice is to apply an NPWT dressing to a grafted area and previously would have applied a VAC (KCI) for 3 days as an inpatient. An estimate of cost savings is based on the rate per night as an inpatient at Bartshealth (£325 per bed day) (Bartshealth Finance Department, Royal London Hospital, UK). There was a potential saving of 24 bed days (£7800) with those patients (n = 8, 1 patient did not need a VAC) receiving a split-thickness skin graft and also would have required VAC therapy if PICO was not available. Considered potential cost savings included continued dressing changes; compensated travel to the clinic (transport cost £22-£76 per patient); and VAC hire and sponges (cost to Bartshealth @£140) to grafted wound.

## DISCUSSION

There has been an expansion in the number of NPWT devices on the market in recent years. The open cell polyurethane foam negative pressure systems, that is, VAC (KCI) and Renasys EZ Plus (Smith and Nephew) are widely used in many hospital settings in managing trauma^12^^,^^13^^,^^16^; pressure ulcers^17^; fistulae^18^^-^20; and elective cases after STSG,^11^^,^^21^ tumour resection,^22^ or above dermal regeneration matrices, including Matriderm (Medskin solutions)^23^ and Integra (LifeSciences Corp).^24^^-^26 Further development of the interface dressing can now achieve complex wound closure with a nonadherent gauze NPWT device instead of a foam dressing, such as the RENASYS-G (Smith and Nephew) based on the method known as Chariker-Jeter system.^12^^,^^27^ To allow better patient management in the home setting, the portable NPWT devices (Acti VAC Therapy System, KCI) and RENASYS GO have their use but are still fairly bulky, noisy and require maintenance of the device while the patient is in the community.^28^^,^^29^ Most recently, a device that utilizes a mechanical spring power instead of electrical power to generate subatmospheric pressure was introduced (Smart Negative Pressure Wound Care System [SNaP, Spira-cur, Inc, Sunnyvale, California]) in the effort to treat refractory chronic wounds in the community.^30^

Recent attempts at an international consensus on the use of NPWT devices take into account wound management and protection, infection management, patient comfort, improved split skin graft take, and costs.^31^^,^^32^ The single use PICO dressing is one that achieves most of the consensus with a steady subatmospheric pressure to the wound bed or graft within the evaluated effective therapeutic range of 50 to 125 mm Hg.^31^^,^^33^^-^36 Evaluating the effects of varying levels of subatmospheric pressure on the rate of granulation tissue formation, scientific research revealed that interstitial fluid pressures beneath the foam dressing does not extend beyond 2 mm into underlying tissue and interstitial fluid pressures became equal to control values within the first millimeter of wound tissue below an NPWT foam dressing. This was irrespective of the applied pressure.^37^ Also, the continuous 70 mm Hg pressure has no contraindication as seen in higher pressures for ischemic wounds^38^ and successfully treated our patient described in case 3 with known peripheral vascular disease. It does not encourage granulation ingrowth into the adsorbent wound contact layer unlike foam sponges,^31^^,^^39^^,^^40^ and consequently all patients tolerated the PICO system with no pain experienced at any dressing changes.

Inpatient NPWT has been used successfully to immobilize the graft material while revascularization takes place instead of conventional dressings.^10^^,^^11^^,^^21^^,^^41^ Studies promoting more ambulant NPWT dressing were still considered “bedside” inpatient treatment.^10^ Several of our patients required SSG to prepared wounds, normally managed as an inpatient with an NPWT device postoperatively. The PICO has revolutionized this management on a purely outpatient basis where in some cases it was applied successfully to prepare the wound bed, succeeded by a day case application of the STSG and PICO and no inpatient bed stay. We estimate approximately a £10,000 bed stay saving due to this alteration in practice (total PICO management for the 10 patients cost £3300). A clinical trial study with cost analysis in Melbourne demonstrated a minimum treatment cost of Aus$3180 for commercial VAC-dressed wounds and inpatient stay: with a net saving of $2603 per patient when using an outpatient ambulant device.^42^

Where we encountered the maximum benefit was that it has the capability of absorbing up to 300 mL of exudate from the wound bed. The 2 cases, where the chyle leak and groin seroma were a major concern to the patient, required up to 3 to 4 dressing changes per day as an inpatient before the PICO application. These chronic exudative wounds are difficult and costly management concerns placing stress on limited resources. Several operative techniques decrease seroma formation in groin dissections,^43^^-^45 but when there is recalcitrant chronic discharge many patients have to be managed with conservative regular dressings. We found the PICO system significantly decreased the exudate quickly over a 7 day period so that dressings could be achieved every 3 to 4 days and then application weekly. The mechanism of reducing persistent lymphorrhea is uncertain, but clinical studies on epidermally applied subatmospheric pressure to surgical wounds or intact skin^46^^-^49 and the observed proliferation of lymph vessels inside and around the vacuumed wound may explain this phenomenum.^50^

Management of a high output chyle leak (>1000 mL/day) can include bed rest, surgically oversewing the area, application of TISSEEL (Baxter Healthcare), transposition of muscle, trial TPN and apply suction, and absorbent dressings to the area.^51^^,^^52^ Our patient received all of this management to control the recalcitrant chyle leakage of up to 4 L a day and the consequential major metabolic disturbances. The chyle leak abated but the wound continued to produce 150 to 220 mL of serous fluid per day from a small neck wound. The PICO was applied epidermally to include this small skin defect and permitted the patient to go home almost free of serous discharge after only 9 days.

There is a holistic approach to the management of more complex wounds keeping in mind every opportunity for the patient to be treated at home and away from potential hospital-acquired infections. People are living longer, with more complex conditions, and are undergoing more extensive surgical procedures; therefore, the numbers with chronic wounds and wound complications continue to increase.^53^ It is also a time of national austerity, and all hospitals are not without their cost improvement programmes. The initial outlay of the dressing cost may appear excessive, but following specific guidelines for the use of this dressing has boosted the departmental savings twofold: reduced patients inpatient stay which resulted in opening up another bed for a further patient admission. The estimated cost improvement was based on how these patients would have been managed without the application of PICO dressing. It can be seen that these PICO patients would have remained inpatients for several more days for either the application of a nonportable NPWT dressing, as this mode of wound management was the care of choice, or that suboptimal simple absorbent dressings wound have continued, prolonged hospital stay, increased costs, and delayed recovery.

## CONCLUSIONS

We have successfully treated a heterogeneous group of surgical and traumatic wounds on an outpatient basis, optimizing their care with the ability to supply a simple home NPWT dressing. It has been clinically evaluated that NPWT dressings have a wide range of beneficial application but their use may be limited by a simple lack of mechanical inpatient or portable devices. These disposable NPWT have significantly optimized our patient care, promoted rapid wound healing, and importantly helped manage costs.

## Figures and Tables

**Table 1:** Twenty-one patients with diverse surgical and traumatic wounds seen in the plastic surgery department for application of the PICO NPWT system on an outpatient basis

					Costs (estimated) £	
Gender	Age	Wound	PICO, n	Application, d	PDC*	PICO
Female	76	Pre-tibial laceration + SSG^†^	3	21	246	360
Female	39	Skin graft to arm	1	7	0	120
Male	40	Lower limb trauma	1	7	0	120
Male^‡^	26	Post-DFSP resection to flank	4	25	820	480
Female	44	Groin dissection seroma	5	31	492	600
Male^‡^	73	Saphenous vein harvest site breakdown + SSG^†^	4	28	328	480
Female^‡^	59	Neck dissection with flap breakdown	5	29	738	600
Female^‡^	57	Neck dissection and right chyle leak	5	24	656	600
Male	49	Groin wound breakdown	3	18	246	360
Female	86	Pre-tibial laceration	4	28	328	480
Male	46	Lower limb trauma	1	7	0	120
Female	39	Hydradenitis axilla + SSG^†^	1	7	1	120
Female	66	Abdominal wound dehiscence	4	25	328	480
Female	40	Skin graft to buttock burn^†^	2	14	164	240
Male	35	SSG to lower limb trauma^†^	1	7	0	120
Female	64	Pretibial laceration + SSG^†^	3	21	246	360
Male	18	SSG to lower limb^†^	1	7	82	120
Male	72	Dog bite to leg SSG	1	7	82	120
Male	56	Wound to lower back e^†^	1	7	0	120
Female^‡^	61	Surgical wound breakdown, breast	7	37	738	840
Female^‡^	43	Seroma post–groin dissection	5	31	246	600
					Total	Total
					£ 5905	£ 7440
					72 visits	62 PICO

SSG indicates split skin graft; NPWT = negative pressure wound therapy; PDC plastic surgery dressing clinic.

*Plastic surgery dressing clinic appointments.

^†^Patients receiving a split skin graft who would have had a 3 day inpatient stay.

^‡^The 6 Patients highlighted as case studies.
